# Working as a Registered Nurse During Menopause—A Multiple Methods Study

**DOI:** 10.1111/jan.70128

**Published:** 2025-08-06

**Authors:** Siranko Heta, Stolt Minna, Hult Marja

**Affiliations:** ^1^ Department of Nursing Science University of Turku Turku Finland; ^2^ Wellbeing Services County of Satakunta Pori Finland; ^3^ South‐Eastern Finland University of Applied Sciences Mikkeli Finland

**Keywords:** menopause, mixed method research, nursing, registered nurse, well‐being at work, working life

## Abstract

**Aim:**

To describe the experiences of Finnish registered nurses aged 45 and over working during menopause.

**Design:**

Multiple methods study.

**Methods:**

The data were collected from Finnish registered nurses aged 45 and over, using two different methods. Quantitative data (*n* = 3487), collected in January 2023, were analysed using descriptive statistical methods. Qualitative data were collected during the summer of 2023 through individual interviews (*n* = 23). The participants were recruited from a survey, where registered nurses (*n* = 3487) who responded to the survey indicated their willingness to participate in the interview study (*n* = 718). Participants for the interviews were selected through random sampling, and interviews were conducted until saturation was reached. The quantitative data were analysed with descriptive statistics, and qualitative data were analysed using inductive content analysis. The results of quantitative and qualitative data were combined in the discussion section.

**Results:**

Limited attention has been given to understanding the menopause and its consequences on the nursing workforce. Menopause remains a taboo topic, with a perceived divide between genders and generations, even within the healthcare sector. However, peer support from female colleagues of a similar age was considered invaluable. During menopause, nurses did not receive sufficient support from their managers or occupational health services, despite experiencing various challenges. Fatigue, for instance, was reported by 76% of nurses aged 45 and over. Nevertheless, nurses continued working despite their symptoms, as taking sick leave was perceived as difficult.

**Conclusion:**

The consequences of menopause on nursing work are not yet sufficiently recognised within workplaces, or by the leadership and occupational health services. Support for nurses working during menopause seems to be insufficient. Open and informed discussions are needed across various levels of society to increase understanding of the problems of working during menopause.

**Implications for the Profession and/or Patient Care:**

The research findings can be used to develop improved occupational health and nursing management practices to support the well‐being of menopausal nurses in the workplace.

**Impact:**

Currently, there is insufficient knowledge about working as a registered nurse during menopause. However, research findings are enhancing our understanding of the impact of menopause on nursing work and the corresponding needs during this period.

**Reporting Method:**

The Standards for Reporting Qualitative Research (SRQR).

**Summary**

- What does this paper contribute to the wider global clinical community?
  ○ Understanding that menopause can affect a nurse's means of coping, particularly in shift work.
  ○ During menopause, job modifications and/or flexibility, such as adjustments to shift work, may be necessary.
  ○ It is essential to normalise discussions about menopause and break the stigma surrounding it to enhance awareness and bridge the gap between genders and generations.

## 1 Introduction and Background

The healthcare sector is facing a global crisis due to a worsening shortage of nurses (WHO 2024), and the situation is similarly concerning in Finland (Tevameri 2021). Registered nurses (RNs), the majority of whom are women, constitute the largest professional group in healthcare. There are approximately 29 million nurses worldwide (WHO 2024). The contribution of RNs is indispensable to the healthcare sector and society as a whole. The nursing workforce is aging; for instance, one‐third of nurses in Finland are expected to retire within the next 10 years (KEVA 2021). As a result, a growing number of women of menopausal age remain active in the profession. This includes an increasing proportion of aging registered nurses whose specific needs—such as those related to menopause—require greater attention and support.

The importance of extending working careers, particularly in the healthcare sector, has become increasingly evident with the aging population. However, RNs often retire several years before the official retirement age due to factors such as work‐related fatigue (Hewko et al. 2019) or health problems (Hidinger et al. 2022). The ability and willingness of RNs to continue working must be supported. Addressing the sustainability gap in public finances requires extending careers, which necessitates raising the retirement age in healthcare and across society more broadly (Ikonen et al. 2019).

Menopause can be defined as the final menstrual period, marking the end of the menstrual cycle. Perimenopause refers to the transitional phase through menopause and includes the time before the final menstrual period as well as the 12 months following it (Cunningham et al. 2025). In this study, menopause is discussed in a broader context, referring to perimenopause, which includes the transitional phase leading up to menopause as well as the time following it. The menopausal transition, including the symptoms, can last, depending on the individual woman, for over a decade (Santoro et al. 2021). Menopause is known to impact women's health and well‐being in various ways. It often causes both mental and physical challenges, including mood swings and fatigue (Harper et al. 2022; O'Neill et al. 2023; Verdonk et al. 2022). Additionally, menopause frequently reduces quality of life, increases sickness absence, and contributes to early retirement (D'Angelo et al. 2023; Verdonk et al. 2022). Furthermore, menopause is known to affect work performance (D'Angelo et al. 2023).

Menopause can also lead to working with reduced work ability (Hashimoto et al. 2020). Furthermore, evidence from prior research indicates that severe menopausal symptoms are associated with reduced work ability (Khajavian et al. 2024). Moreover, studies in industrial settings, such as manufacturing, suggest that night shifts can exacerbate menopausal symptoms (Sawamoto et al. 2024).

The challenges posed by menopause in the workplace, including those from a management perspective (Verdonk et al. 2022), have not yet been sufficiently recognised or addressed, despite existing recommendations aimed at supporting work ability in the workplace (Rees et al. 2021). Previous research emphasises the need for tailored occupational health and organisational practices to support women's career sustainability, particularly during menopause (Nurmeksela et al. 2023; Viotti et al. 2020). Working during menopause, especially in nursing—a profession characterised by shift work and tasks requiring high levels of precision—has received limited research attention. There is a notable lack of in‐depth knowledge regarding the experiences of RNs working during the menopausal transition and menopause itself. This is despite menopause being widely recognised as a significant phase in a woman's life that impacts both health and work ability. Given the female‐dominated nature of the nursing profession and the aging workforce, it is crucial to investigate menopause and its potential effects on RNs' work. This study seeks to address this knowledge gap by exploring the needs and experiences of RNs during menopause in their work and workplace. These insights could provide occupational health services and nursing management with information to help develop practices better suited to supporting employees during this critical phase of working life. This study focuses on registered nurses and their experiences of menopause in the workplace.

### 1.1 Aim

The aim of the study was to describe the experiences of registered nurses aged 45 and over regarding working during menopause.

**The research questions were:**

1. What is the prevalence of health problems among registered nurses aged 45 and over?
2. How do registered nurses describe working during menopause?

## 2 Methods/Methodology

### 2.1 Design

The study employed a multiple methods approach. In the first phase, the quantitative component investigated the prevalence of health problems among RNs aged 45 and over. Based on occupational psychology, workforce demographics, and age‐related cognitive and physical studies, the age range of “aging workers” is generally considered 45–65 (see e.g., Burmeister et al. 2020). The second phase, the qualitative component, provided deeper insights into the experiences of RNs aged 45 and over working in healthcare during menopause. The quantitative and qualitative data were analysed independently, and the findings were integrated in the discussion chapter.

### 2.2 Study Setting and Recruitment

Recruitment for the survey was conducted in January 2023, in collaboration with a Finnish trade union representing healthcare workers. The trade union included an invitation to participate in the survey in their newsletter, which was sent to all members (*N* = 100,000). A total of 4574 trade union members responded. However, we could not calculate a response rate, as it was not possible to determine how many members actually opened the newsletter. Among the respondents, 3487 were registered nurses, and 718 indicated their willingness to participate in the interview study. From this group, 70 participants were randomly selected using a table of random numbers. The selection was limited to registered nurses aged 45 and over. Ultimately, 23 of the invited nurses participated in the interviews. Although we were prepared to invite additional participants if necessary, data saturation was reached after interviewing 23 nurses. The participants represented a diverse range of healthcare organisations from across Finland.

### 2.3 Inclusion Criteria

The inclusion criteria for the quantitative study were being aged 45 and over, having the education of a RN, and being female. The qualitative data collection followed the same inclusion criteria.

### 2.4 Data Collection

#### 2.4.1 Quantitative Data Collection

The members of a healthcare workers' trade union received an invitation to participate a survey on working life quality and health in the newsletter. The data were collected by an online tool, Webropol. This study included a set of questions about health problems (*n* = 10), with dichotomous response options of 1 = yes and 0 = no. The questions addressed for example the following: headache, depression, general fatigue, insomnia or sleep disturbances. The self‐rated health (SRH) was assessed with one question about the current health status. The response options ranged from 1 (Poor) to 5 (Very good). The responses from 4 to 5 indicated good health, whereas responses below 4 indicated decreased health.

#### 2.4.2 Qualitative Data Collection

For the qualitative interview study, a semi‐structured interview guide was developed, following recommendations for semi‐structured interview frameworks (Kallio et al. 2016). The interview guide (File S1) was evaluated by three researchers and tested with one woman of menopausal age. After testing, the wording of the questions was refined and finalised. The final interview guide included questions under two main themes: aging and working life, and menopause and working life. The data generated from the aging and working life theme will be reported separately elsewhere. Two test interviews were conducted, resulting in no changes to the interview guide; therefore, these interviews were included in the data. The interviews were conducted between May and September 2023 by one researcher (HS) via MS Teams or by telephone. All the interviews were recorded. The interviews continued until no new topics emerged, indicating that data saturation was reached (*n* = 23). The interviews lasted between 35 and 84 min, with a mean duration of 60.5 min. The interviews resulted in a total of 23 h and 12 min of material. The data were transcribed into 305 pages using Calibri font, size 12, with 1.5 line spacing.

### 2.5 Data Analysis

The quantitative data were analysed statistically with SPSS version 29. Descriptive statistics (percentages, ranges, means and standard deviations) were used to describe the study participants and their health status.

A qualitative component was chosen to facilitate a deep and comprehensive understanding of the research topic. The qualitative data were analysed using inductive content analysis. The data analysis followed a similar procedure to that described by Elo and Kyngäs (2008). The analysis began with familiarisation with the entire dataset by reading it through multiple times. After familiarisation, a unit of analysis was selected based on the entirety of thoughts expressed. Meaning units were then extracted from the transcribed data that responded to the research question. At this stage, it was also confirmed that the data provided answers to the research question. Next, the original expressions were condensed by removing unnecessary filler words and converting spoken language into written language (Elo and Kyngäs 2008). Since the unit of analysis was a meaning unit, each original expression could be condensed into one or more simplified expressions, with each simplification containing only one core idea.

After condensing the original expressions, the data were organised by marking different concepts and creating tentative headings. These codes (*n* = 206) were collected into an Excel spreadsheet, where they were freely grouped. Similarities and differences in the original data were then examined. Simplified expressions with similar content were grouped into the same subcategory, and subcategories were named to reflect their specific content (Elo and Kyngäs 2008). Subcategories were subsequently combined into broader categories by grouping together those with similar content (Table 1). A total of 15 categories were identified and named according to their content. The abstraction process continued by forming three main categories from these broader categories: Understanding menopause in working life, Well‐being at work during menopause, and Menopausal symptoms. The abstraction was further refined by creating one overarching category: Working as a registered nurse during menopause.

### 2.6 Ethical Considerations

The study was conducted in accordance with good ethical practices (ALLEA 2023). In Finland, ethics committee approval is not required for interview studies involving voluntary, competent, and non‐vulnerable adults (TENK 2019). Before collecting quantitative data, research permission was obtained from the trade union that facilitated the survey. Participants in the interview study were given the opportunity to review a written research information sheet in advance, and the voluntary nature of the study, as well as key details, were also explained orally before requesting consent. Participants provided verbally informed consent for the study, which was recorded. The pseudonymised data were securely stored on a password‐protected server at University of Turku, with access restricted to the principal researcher (ALLEA 2023).

### 2.7 Rigour and Reflexivity

The study's trustworthiness was supported through dependability, confirmability, and transferability (Ahmed 2024). Efforts were made to reduce interpretation bias caused by preconceptions through continuous discussions within the research team throughout the process. To ensure dependability and transparency, an explanation of the analysis process was written, and the justification for the analysis was supported by including direct quotes from the data in the results. The entire research process was described as precisely and comprehensively as possible to enhance confirmability.

## 3 Findings

### 3.1 Description of Participants

#### 3.1.1 Characteristics of Participants

The mean age of the survey participants, all of whom were registered nurses (*n* = 3487), was 54 (range 45 and 67 years). The mean age of the interviewees (*n* = 23) was 57 (range: 48–66 years). One of the interviewees assessed themselves as being in the premenopausal phase, six in the perimenopausal phase, 15 in the postmenopausal phase, and one was unable to determine their phase.

All participants had a bachelor's degree in nursing, and some had also completed vocational education or a higher university degree. All participants were employed in permanent positions: 17 worked in the public sector, five in the private sector, and one in another type of organisation.

### 3.2 Prevalence of Health Problems Among RNs Aged 45 and Over

Quantitative data analysis revealed that 45% of RNs reported a decreased health. Musculoskeletal pain in the neck or shoulder was experienced by 78% of RNs, while 76% reported general fatigue. Headaches and/or eye pain were reported by 69%, as were muscle pains in the lower body. Sleep disturbances were noted by 67% of RNs. Additionally, 59% experienced back pain, 35% reported abdominal pain, 20% had breathing difficulties, and 19% indicated symptoms of depression or anxiety. Injuries or wounds were reported by 10% of RNs.

### 3.3 RNs' Experiences of Working During Menopause

**TABLE 1:** Categories with sub‐categories.

Categories	Sub‐categories
Understanding menopause in working life	Awareness of menopause
	Attitudes toward menopause
	Discussing Menopause
Well‐being at work during menopause	Shift work
	Role of occupational healthcare
	Manager support
	Peer support
Menopausal symptoms	Difficult periods
	Hot flushes and night sweats
	Headache and migraine
	Sleep problems
	Cognitive difficulties
	Changes in mental health and mood
	Social challenges
	Working with symptoms

#### 3.3.1 Understanding Menopause in Working Life

Understanding of menopause in working life consisted of three categories: awareness of menopause, attitudes toward menopause, and discussing menopause.

##### 3.3.1.1 Awareness of Menopause

The nurses indicated that, in general, understanding and acknowledgement of menopause is limited at the healthcare workplaces. It was perceived that colleagues, managers, and even the participants themselves had too little knowledge about menopause, and there is not enough awareness of how it can affect individuals. The nurses felt that especially managers lacked sufficient knowledge.

The understanding of menopause is almost nonexistent, which seems absurd considering it's a female‐dominated field. Everyone goes through it, yet it's not treated as a natural part of life there. Everyone understands when someone is pregnant because it's visible, and it's clear that she may not have as much energy and that her moods might fluctuate. But when you go through menopause… people don't know how to handle it. (RN 18)

On the other hand, nurses' self‐awareness varied; although it was felt that their healthcare education provided them with more knowledge than the average person. It was also perceived that colleagues and managers who had already experienced menopause had a better understanding of its effects compared to those who had not gone through it themselves.

I feel that my competence is above average because I have an educational background in public health nursing, and I've also had a long career as a nursing manager, which has kept me in touch with these issues, along with my own personal experience. (RN1)

The nurses felt that increasing awareness of menopause is important because it would enhance understanding of its effects on individuals and their ability to work. It was described that greater understanding of menopause would make it easier to cope and to discuss menopause openly. Increased understanding was also linked to the idea that challenges faced during menopause would be better recognised and addressed within the workplace.

##### 3.3.1.2 The Attitudes Toward Menopause

Menopause evoked diverse feelings among nurses, from relief to shame. On the one hand, menopause was seen as a normal part of life, and on the other, it caused considerable shame; this shame was thought to arise, for example, from the stigma of being an aging woman or a perceived decline in performance. Nurses also highlighted that the menopausal phase is a temporary stage in a woman's life. Menopause was generally seen as a taboo topic, even in the healthcare sector, despite it being a part of every aging woman's life.

I myself felt a sense of shame for not being at my most energetic or sharp, and it made me feel embarrassed. (RN 16)

The nurses felt that there is a sort of gap between generations and genders as regards attitudes toward menopause. The gap was explained, for example, by a lack of understanding and knowledge, which led to insufficient recognition of the fatigue, or other symptoms experienced by those going through menopause.

There is perhaps a kind of gap with younger people; they don't understand it yet. (RN 19)

Menopause and its symptoms were felt to be mocked, mostly by male colleagues and belittled, especially by younger colleagues. The nurses shared that joking about the ‘granny disease’ felt hurtful. Teasing from male colleagues on this topic, in particular, caused distress. In contrast, joking with women of the same age was perceived as good‐natured humour.

I do think it's often seen as a bit of a joke, like ‘Oh, she's being cranky again, she must be going through menopause.’ It's somehow belittled and, in a certain sense, even mocked. Quite often, it's dismissed as just some ‘granny disease’, with comments like ‘try to calm down now’. (RN 5)

##### 3.3.1.3 Discussing Menopause

Talking about menopause was generally perceived as difficult in the workplace. Menopause was thought to be associated with many negative stereotypes, which were believed to contribute to the reluctance to discuss the topic. Nurses felt that menopause carries an unpleasant stigma.

One colleague going through menopause received quite a bit of negative feedback. She was very irritable and angry, and had conflicts with almost everyone. As an exaggerated example of menopause, this likely contributed to a lot of negative perceptions about it. This is exactly how no one wants menopause to be seen, which might be why people don't want to talk about it. They don't want to be labeled as a ‘crazy woman’, a ‘menopausal woman’. (RN 7)

However, whether menopause was discussed in the workplace depended entirely on the specific work community and whether there were colleagues going through the same stage of life. At best, in some work communities, menopause and the challenges it brought, such as sleep difficulties, could be discussed quite openly at the coffee table.

Often at the morning coffee table, there's talk like, ‘I don't know how today will go, my head is in a fog after a night like that’. (RN 15)

Conversely, in some workplaces, discussing menopause was considered impossible. Nurses mentioned that they did not want to talk about menopause in the presence of male colleagues. Talking with women of the same age felt easier for nurses.

Since we have a workplace with both men and women, it's not talked about much. I think if it were an all‐female workplace with more women of that age, it would be discussed more. (RN 2)

It depends on the manager, whether menopause and the challenges it causes were broached with them. Generally speaking, female managers of the same age or older were perceived as the safest to talk to about it. Discussing menopause with male managers was difficult for nurses. However, even in this case, a trusting manager‐employee relationship emerged as the most important factor.

It doesn't matter if I had period pain or anything else, I still wouldn't go and talk to them about it. But I think that if there was a really good relationship, a good trusting relationship, then I don't see it being an issue whether they're a man or a woman. (RN 5)

#### 3.3.2 Well‐Being at Work During Menopause

Well‐being at work during menopause is constructed of four categories: shift work, the role of occupational healthcare, manager support, and peer support.

##### 3.3.2.1 Shift Work

The nurses felt that the importance of a consistent schedule increases during menopause. A more consistent life was felt to bring much‐needed stability and, through that, potentially better well‐being.

It probably would have made life easier, bringing more balance and regularity, and perhaps I would have felt better at that point. (RN 21)

Shift work, especially night shifts, was perceived as very challenging during menopause. Nurses described recovery from night shifts as particularly demanding. Much more time was needed for recovery from night shifts than the work schedule allowed. Those who had the opportunity to skip night shifts during menopause felt it significantly helped their coping and well‐being.

Especially with night shifts, recovering isn't as easy as it was when I was younger. Sleeping during the day isn't as straightforward, so sometimes, even after a full day, I still haven't fully recovered from those night shifts. (RN 16)

Shift planning that considers individual needs was generally seen as important during menopause. Nurses felt that their needs and preferences were well accommodated in their workplace, which was perceived to positively impact their ability to cope at work. On the other hand, it was also felt that, despite promises, their requests were neither listened to nor implemented.

It helped in that it's easier to sleep at night than during the day… it definitely made a big difference. (RN 18)

Additionally, nurses wondered whether greater flexibility could be implemented in healthcare workplaces as well. For example, they questioned why morning shifts have to start so early for everyone. More flexibility in the form of work adjustments was desired, such as the option to take on lighter duties when needed. In some workplaces, nurses were given the option to work part‐time, which was seen as supportive of well‐being. However, the option for part‐time work was not always available, and this was perceived as unfair. Situations were also identified where the employer does offer flexibility, such as allowing a two‐hour window for starting work. However, this option was often not utilised, as most employees chose to start as early as possible to finish their workday earlier.

What I've noticed in some workplaces is that there's been flexibility, allowing a start time between 6:00 and 8:00, but everyone tries to come in at 6:00 to leave earlier. (RN 8)

##### 3.3.2.2 The Role of Occupational Healthcare

It was generally felt that the role of occupational healthcare increased during menopause. However, menopause was not recognised in any meaningful way within occupational healthcare. Nurses hoped for a greater role from occupational health services in supporting work ability during menopause and in preventive measures. By a preventive approach, nurses referred to actions such as occupational health proactively reaching out to individuals of a certain age to provide information and support in advance about how menopause might impact well‐being at work and what considerations individuals should keep in mind. Additionally, the possibility of meeting with a gynaecologist through occupational health services was also desired. Quite simply, there was an expectation that occupational healthcare professionals would have more knowledge and courage to raise menopause both at the individual level and within organisations.

If occupational health services have the resources and are given the opportunity, they could take a much more proactive approach in many areas… they could actively reach out to work units and communities to provide information. For instance, in matters like sleep management, they could serve as the primary source of information. (RN 11)

Nurses also felt that the passive role of occupational health in supporting well‐being during menopause is partly due to insufficient resources. They reported that it was generally difficult to get an appointment with occupational health services, particularly within a reasonable timeframe. On the other hand, it was felt that if they sought help from occupational healthcare on their own, they did receive assistance, but not specifically related to menopause.

Well, occupational health services would definitely be the place to go, and that's probably where I'd start looking for help with this condition… But it might take several months to get an appointment for issues like this. (RN 15)

##### 3.3.2.3 Manager Support

Generally, nurses hoped to receive support from managers during menopause. The support they sought included genuine interest in employees' well‐being, open discussions, and concrete actions when needed. Nurses felt that receiving such support could have a significant impact on their well‐being. They mentioned that support from their manager during menopause was evident in their genuine interest in the employee's well‐being and always having time to discuss any issues.

She approaches things very sensitively, inviting you to come talk if she notices something, or if someone asks to speak with her, she always makes time. (RN 19)

Nurses felt that menopause is a challenging topic for many managers as well. Usually, managers do not bring up the subject, which nurses suspect is due to its personal nature. There are also perceived differences in the support provided by managers, depending on the manager's gender and age. They felt that slightly more senior female managers had the understanding needed to provide the necessary support.

I can imagine‐my manager is a man, and I'm sure he'd rather carry a bag of rocks than have a conversation about menopause. That's for sure. (RN 16)

##### 3.3.2.4 Peer Support

Peer support was often felt to be essential and made a significant impact on workplace well‐being during menopause. The work community's understanding of working during menopause was variable. Nurses found it easy to share experiences with those in a similar life stage, receiving support and understanding from them readily. The shared experience and a humorous approach to menopause‐related symptoms helped nurses cope better at work. Some described it as a relief to have someone who understood why, at times, they just do not have the energy. In contrast, some did not find peer support in the workplace essential, placing greater value on their personal circle of friends outside of work. It also depended entirely on the workplace and its characteristics as to what kind of peer support was available.

We always encouraged each other and felt like we knew and understood one another. (RN 18)

#### 3.3.3 Menopausal Symptoms

Nurses described a variety of menopausal symptoms that affected their work in healthcare. The symptoms were divided into eight categories: difficult periods, hot flushes and night sweats, headache and migraine, sleep problems, cognitive difficulties, changes in mental health and mood, social challenges, and working with symptoms.

##### 3.3.3.1 Difficult Periods

During menopause, heavy menstrual flooding was felt to complicate work, as there was often no opportunity to change menstrual products as needed, and no product's absorbency was sufficient for more than a short period. The irregular periods also caused occasional stress and frustration, as it could start when least expected. Nurses also felt that their periods had become more severe than before, and they needed to keep pain relievers on hand in case of menstrual pain.

You never know when your period will start. And when it does, the bleeding is really heavy, so you can't really relax‐you're always on alert, wondering if there's any leakage, where the nearest restroom is, and when your breaks are. (RN 3)

##### 3.3.3.2 Hot Flushes and Night Sweats

Hot flushes and night sweats were often felt to make life more difficult. The heat in the body was described as overwhelming and sweating was also considered an embarrassing, visible symptom, leading to concerns that others might think they were unclean. The discomfort of sweating was partly due to the fact that it was uncontrollable and could not be hidden. Sweating also posed practical challenges, as some had to consider how many changes of clothes to pack for the workday.

The amount of night sweats was probably the biggest issue–it disrupted all sleep. You'd have to change your pajamas, then lie back down on damp sheets, wondering whether to try to sleep or change the sheets. It really took a huge toll during that time. (RN 16)

##### 3.3.3.3 Headache and Migraine

Headaches and migraines became more frequent for nurses during menopause. It was described that headaches or migraine attacks were linked to fatigue or poor sleep, while for others long work shifts exacerbated these issues.

After long work shifts, I get those attacks. For years, I didn't have them, but in the past couple of years, likely related to menopause, they've reactivated. (RN 19)

##### 3.3.3.4 Sleep Problems

Sleeping difficulties, which were described as trouble falling asleep, lighter‐than‐normal sleep, and frequent nighttime waking, were generally described as one of the most challenging effects of menopause on workplace well‐being. Sleeping difficulties led to nurses feeling constantly tired. The nurses described that, at its worst, fatigue could have led to work disability and early retirement due to health reasons. Nurses shared that they managed at work but were exhausted by the time they got home. They felt that their family suffered the most from the menopause, as the exhaustion from getting through the workday was often taken out at home, for example, on their spouse. Sleep difficulties were affected by various symptoms, such as night sweats and restless legs.

I was pretty much‐no, not just pretty much, I was completely worn out from the sleeping problems, exhausted and totally tangled up. (RN 11)

##### 3.3.3.5 Cognitive Difficulties

Nurses described having difficulties concentrating at work due to menopause. Some made mistakes at work, which increased the stress they experienced. Having difficulties with their memory were also mentioned. Those struggling with memory had started using checklists, with some feeling that every task needed to be written down to ensure it was completed. Cognitive challenges were largely attributed to sleeping difficulties.

And then, noticing that your memory slips and you're less able to concentrate—it really does affect you. (RN 23)

##### 3.3.3.6 Changes in Mental Health and Mood

Menopause was described as causing mood changes and increased sensitivity, which manifested, for example, as heightened tearfulness. Nurses described feeling irritable and short‐tempered due to menopause.

I've definitely noticed that I've become more sensitive… I tend to take criticism very personally now, and I've always been quick to laugh and cry, but maybe the tearfulness has become even more intense. (RN 19)

Nurses experienced stress and uncertainty due to fatigue and forgetfulness. At its worst, the mental distress during menopause was so overwhelming that some felt they could no longer cope. At such times, the state was described as feeling broken.

I've been at a point where I was so broken that, to be honest, I was almost at the verge of wanting to jump off something. (RN 11)

##### 3.3.3.7 Social Challenges

Menopause was felt to impact social relationships at work. For example, the time was also described as one in which all workplace relationships became strained due to the mental challenges caused by menopause. Nurses reported being less able to manage customer meetings as effectively as before and, when possible, needing to plan more carefully how many customers they could engage with fully in a day.

You really have to consider how many customers you can handle in a day to genuinely be able to help. (RN 5)

##### 3.3.3.8 Working With Symptoms

Those going through or having experienced menopause described working with the symptoms. The nurses mentioned that, for some reason, they did not want to stay at home even when experiencing symptoms but rather chose to go to work. They reflected on this decision later, questioning why they pushed themselves and acknowledging that it would have been better to stay home and rest when not feeling well.

Well, I did have migraines too, but I never took time off because of it, not once. (RN 20)

Working during menopause was described, for example, as “working persistently, even though they felt they could not.”

Then there was the sleep, of course—it was sometimes so poor that I just had to push through the workday. (RN 7)

## 4 Discussion

The research findings support previous studies suggesting that menopausal symptoms have a negative impact on work (Alzueta et al. 2024; Steffan and Potočnik 2023). The study provided new insights into RNs' experiences of working during menopause. The findings indicated that the level of understanding of menopause in healthcare workplaces varied. However, overall, menopause remained a complex and challenging topic. A gap between knowledge and attitudes toward menopause was observed, influenced by both gender (Verdonk et al. 2022) and generational differences. The challenging nature of the topic was reflected in the fact that menopause was still perceived as a sensitive subject for discussion in workplaces (Verdonk et al. 2022). The findings also suggest that its significance within work communities and management practices remains insufficiently understood and acknowledged. Previous research supports these findings. For example, Cronin et al. (2024) found that menopause is poorly understood, and employees in the healthcare sector are often reluctant to discuss it with their managers.

However, the lack of recognition and understanding of menopause and its impact on work, particularly in the context of nursing, was unexpected. One might assume that nurses, given their healthcare education, would be more likely to recognise the signs and symptoms of menopause. Verdonk et al. (2022) also described a situation in which even healthcare professionals fail to recognise menopause as a life stage, which can have various negative consequences, for example on the experience of symptoms or feelings of insecurity. Additionally, the observed gap in attitudes and understanding between genders and generations was an unexpected finding, as ageing‐related issues would be expected to be an integral part of healthcare professionals' expertise. On the other hand, older generations are known to approach situations like menopause with more shame or embarrassment than younger generations, as it was not considered appropriate to discuss such topics openly in settings like the home in the past (Wood et al. 2025). Though, one would expect healthcare professionals to possess not only the necessary knowledge and expertise but also the capacity for empathetic interactions with colleagues, recognising the sensitivity of menopause. The sense of shame described by nurses was linked, for example, to a perceived decline in their energy levels during menopause. Being subjected to ridicule, such as the derogatory term ‘granny disease’ mentioned by nurses, likely increases rather than alleviates this shame. Previous research has shown that menopause is perceived as stigmatising, which makes it difficult to discuss in the workplace, as individuals seek to avoid being labelled (Wood et al. 2025; Verdonk et al. 2022). Reducing the stigma surrounding this natural life stage could be facilitated by discussing menopause in a neutral and respectful manner, free from judgement. However, addressing this issue requires a broader societal shift in attitudes toward ageing women, as well as increased awareness and understanding of menopause among healthcare professionals and their managers (Cowell et al. 2024).

Well‐being at work during menopause was perceived as needing support both in the form of job modifications and through assistance from managers and occupational health services. The study findings indicated that shift work, particularly night shifts, posed challenges for nurses during menopause (Sawamoto et al. 2024). The importance of maintaining a regular schedule was especially emphasised when sleep difficulties became more prevalent (Rees et al. 2021). However, the ability of workplaces to provide effective solutions varied. Employers who offered flexibility, such as the possibility of temporarily opting out of night shifts during menopause, were highly valued by employees. The results also highlighted the need for greater fairness and consistency, as nurses reported experiencing inequitable practices at certain workplaces. Previous research similarly underscores the crucial role of workplace flexibility in supporting women during menopause (O'Neill et al. 2023).

Various health problems and menopausal symptoms affected nurses in their work, which is also supported by previous research on the topic (Vanderzalm et al. 2023). The quantitative findings of this study indicated that nearly half of nurses aged 45 and over reported a decline in their health. Compared to the 2023 OECD Health at a Glance report (OECD 2023), the overall health of Finnish registered nurses aged 45 and over appears to be poorer than that of Finnish women in the same age group. According to OECD data, 67% of Finnish women aged 45 and over in 2023 rated their health as good or very good, whereas in this study, nearly half of the participants reported a decline in their health based on quantitative findings. In comparison to other OECD countries, the poorest health within this comparison group was observed in Latvia, where 39.8% rated their health as good or very good (OECD 2023). The best health experience was found in Greece, where 84.3% of the comparison group rated their health as good or very good.

The results suggest that factors such as headaches and sleep disturbances may contribute to the decline in perceived health and coping at work (Verdonk et al. 2022). Sleep difficulties and fatigue were particularly prevalent in this age group: quantitative analysis revealed that 76% of nurses experienced fatigue, while 67% reported sleep difficulties. These findings align with descriptions provided by nurses who participated in the qualitative interviews. Nurses described experiencing severe fatigue, often due to difficulties falling asleep or frequent nighttime awakenings (Vanderzalm et al. 2023).

Nurses perform highly demanding work that requires a continuous focus, and those who participated in the interviews described this as particularly challenging during menopause. Memory impairments associated with menopause also emerged as a significant challenge in cognitively demanding roles (O'Neill et al. 2023; Vanderzalm et al. 2023). Greater attention should be directed towards addressing the cognitive challenges experienced by menopausal nurses to reduce additional stress and anxiety associated with concerns about potential memory lapses while performing professional responsibilities.

Nurses described fatigue as being extremely challenging at times and reported relying solely on willpower to continue working. Despite experiencing symptoms, nurses reported continuing to work, with a high threshold for taking sick leave even when feeling unwell. International research indicates that menopausal symptoms frequently lead to sick leave. In the study by Cronin et al. (2024), over 60% of participants reported that during the previous 4 weeks they had missed work or shortened their workdays due to menopausal symptoms. Variations in the sick leave behaviour of Finnish nurses were observed in this study, emphasising the need for further research. Previous studies have demonstrated that presenteeism—working while unwell—is associated with both deteriorating health and reduced productivity (Lui and Johnston 2019). It would be important to identify if a nurse is working with a reduced capacity.

Menopausal symptoms appeared to create a cyclical pattern in which multiple factors interacted simultaneously, exacerbating challenges and negatively impacting overall health and well‐being (Harper et al. 2022; Verdonk et al. 2022). For instance, quantitative findings indicated that three‐quarters of nurses experienced headaches, and even more suffered from severe fatigue. In interviews, nurses described how sleep disturbances led to persistent exhaustion, which, in turn, impaired their ability to concentrate. This decline in attentiveness further increased stress, particularly in a profession that demands sustained focus and precision (Vanderzalm et al. 2023). Fatigue also made patient interactions more burdensome. Furthermore, the nature of nursing work made taking breaks difficult, which increased stress, particularly for those experiencing heavy and irregular menstruation. Nurses had to continuously ensure they had adequate menstrual protection and plan to be close to the nearest restroom. Excessive sweating and hot flashes further complicated clothing choices for work, requiring nurses to bring extra clothing to accommodate temperature fluctuations throughout their shifts.

Working as a nurse during menopause, as discussed in this study, is a complex phenomenon that requires further attention to enhance understanding and provide tangible support for managing work during this life stage. Since menopause is a temporary but usually a long phase of several years in a woman's life, there is a critical need to better support nurses during menopause so that they can sustain their careers both during and after this transition, especially given that menopausal symptoms are known to decrease work ability (Khajavian et al. 2024).

### 4.1 Strengths and Limitations

The strengths of this study include its sample sizes: 3487 participants in the quantitative study and 23 in the qualitative study. These sample sizes were sufficient to effectively describe the participants' backgrounds and their experiences of working as RNs aged 45 and over. Additionally, data saturation was achieved in the qualitative study. A key strength of this study is that all three researchers, who have educational backgrounds in healthcare and practical experience in the healthcare sector, participated in the data analysis. This involvement ensures a strong contextual understanding of the research topic (Berger 2015).

The limitations of this study include the connection issues in Teams, which disrupted the flow of a few interviews, as well as the cultural sensitivity of the topic, as attitudes toward menopause may vary across countries. Moreover, due to the cross‐sectional design of the quantitative data, we were unable to examine changes in the health experiences of RNs aged 45 and over. Therefore, longitudinal studies are required to understand which factors influence health perceptions over extended periods.

### 4.2 Implications for Policy and Practice and Recommendations for Further Research

Based on the findings of this study, further research is needed to determine how common it is for nurses to work with reduced work ability during menopause. Additionally, further studies are required to explore how healthcare work environments, where tasks are often precision‐demanding and shift‐based, could be adapted, along with work practices, to better support employees during menopause.

The research suggests that findings can be applied to improve workplace practices in the healthcare sector to better support employees during menopause. The need for managerial support was clear, but the sensitivity of the topic created challenges. Furthermore, occupational health services were seen as insufficient in providing menopause support, likely due to resource limitations. This issue needs further exploration to develop strategies for better collaboration between managers and occupational health services to support employees through the menopausal transition.

## 5 Conclusion

Menopause and its impact on work remain significantly underrecognized within the nursing sector, despite its profound implications for employee well‐being and performance. Nurses often receive minimal support in managing the challenges of menopause, both from occupational health services and their direct supervisors. As a result, many continue working in demanding, shift‐based roles even as they experience symptoms that can reduce their work ability—symptoms that may warrant job adjustments or increased flexibility. This lack of tailored support not only affects individual nurses but also has broader implications for workforce sustainability. To address this gap, targeted interventions are urgently needed to empower nurses, promote healthier work environments, and ensure they can navigate this life stage with resilience and dignity.

At the societal level, there is an urgent need to foster open, informed conversations about menopause, accompanied by greater awareness and understanding. Normalising menopause as a natural stage in every woman's life is essential to breaking down stigma and promoting empathy. Increased awareness can also lead to more thoughtful consideration of workplace accommodations for menopausal employees. Although menopause is a temporary phase that may call for specific support measures, it neither diminishes a woman's value in the workplace nor permanently affects her performance. By framing menopause as a manageable transition, organisations can implement supportive strategies that help employees maintain their well‐being and productivity—both during this phase and well beyond it.

## Author Contributions

All authors have agreed on the final version and meet at least one of the following criteria (recommended by the ICMJE [http://www.icmje.org/recommendations/]): Substantial contributions to conception and design, acquisition of data, or analysis and interpretation of data; drafting the article or revising it critically for important intellectual content.

## Conflicts of Interest

The authors declare no conflicts of interest.

## Supporting information


**Data S1:** jan70128‐sup‐0001‐Supinfo.docx.

**Data S2:** jan70128‐sup‐0002‐Supinfo.docx.

## Data Availability

The data are available upon a reasonable request.
