# Supplementary material for: Working as a Registered Nurse During Menopause—A Multiple Methods Study
Source: J Adv Nurs. 2025 Aug 6;82(5):5419–29. doi: 10.1111/jan.70128 (PMC13069225; doi:10.1111/jan.70128)
Supplement: Supplementary file 2 — Data S2: jan70128‐sup‐0002‐Supinfo.docx. [file JAN-82-5419-s001.docx]

**Supplementary file 1**

**Interview guide**

**Opening discussion**

- What made you interested in this study?
- What thoughts or feelings does the study’s topic evoke in you?

**Experiences and perceptions of menopause**

- What thoughts or feelings does menopause evoke in you personally?
- In your experience, how are menopause / menopausal women viewed in society?
- In your experience, how are menopause / menopausal women viewed in the workplace?
- How is menopause discussed at your workplace or within your work community?
- What experiences do you have—or know others to have—of bringing up menopause within the work community, with a supervisor, or with occupational health services?

**Knowledge, information needs, and working during menopause**

- How would you describe your own knowledge or understanding of menopause and its effects on wellbeing, health, and work ability?
- What kind of information would you like about the effects of menopause on wellbeing, health, and work ability?
- What experiences do you have—or know others to have—of working during menopause?
- What is it like to perform nursing work during menopause?
- In what ways has menopause affected your wellbeing, health, or work ability?
- Which factors are important for you in maintaining or improving your work ability during menopause?

**Leadership and occupational health**

- How is menopause taken into account in leadership/management?
- How would you describe nurse managers’ and supervisors’ understanding of menopause and working during menopause?
- How would you like menopause to be considered in leadership/management?
- How is menopause addressed by occupational health services?
- How would you like menopause to be addressed by occupational health services?

**Closing discussion**

- Is there anything else you would like to share about ageing, menopause, or working during this life stage?
